# Picropodophyllin and sorafenib synergistically suppress the proliferation and motility of hepatocellular carcinoma cells

**DOI:** 10.3892/ol.2014.2484

**Published:** 2014-08-27

**Authors:** MINORU TOMIZAWA, FUMINOBU SHINOZAKI, YASUFUMI MOTOYOSHI, TAKAO SUGIYAMA, SHIGENORI YAMAMOTO, MAKOTO SUEISHI

**Affiliations:** 1 Department of Gastroenterology, National Hospital Organization, Shimoshizu Hospital, Yotsukaido, Chiba 284-0003, Japan; 2 Department of Radiology, National Hospital Organization, Shimoshizu Hospital, Yotsukaido, Chiba 284-0003, Japan; 3 Department of Neurology, National Hospital Organization, Shimoshizu Hospital, Yotsukaido, Chiba 284-0003, Japan; 4 Department of Rheumatology, National Hospital Organization, Shimoshizu Hospital, Yotsukaido, Chiba 284-0003, Japan; 5 Department of Pediatrics, National Hospital Organization, Shimoshizu Hospital, Yotsukaido, Chiba 284-0003, Japan

**Keywords:** insulin-like growth factor 1 receptor, scratch assay

## Abstract

Resistance is one limitation of sorafenib in the treatment of hepatocellular carcinoma (HCC). Insulin-like growth factor-1 receptor (IGF-1R) is involved in cancer cell proliferation. To assess the potential synergistic antitumor effects of picropodophyllin (PPP), an IGF-1R inhibitor, HLF and PLC/PRL/5, HCC cells were treated with PPP alone or PPP in combination with sorafenib, a multikinase inhibitor. Normal human umbilical vein endothelial cells (HUVECs) were also used to analyze the antiangiogenic effects of the drugs. HCC cells and HUVECs were cultured on 96-well plates, and then treated with PPP, with and without the addition of sorafenib. A 3-(4,5-dimethylthiazol-2-yl)-5-(3-carboxymethoxyphenyl)-2-(4-sulfophenyl)-2H-tetrazolium inner salt assay and hematoxylin and eosin staining were then performed 48 h later. The HCC cells were also analyzed using scratch assays and hematoxylin and eosin staining after 48 h. The proliferation of HLF, PLC/PRF/5 and HUVEC cells was suppressed by the combination of 0.2 μM PPP and 3 μM sorafenib more effectively than by 10 μM sorafenib alone. The motility of HLF and PLC/PRF/5 cells was also suppressed to a greater extent with the combination of PPP at 0.2 μM and sorafenib at 3 μM than with sorafenib at 10 μM alone. The cells that had been treated with 0.2 μM PPP and 3 μM sorafenib also exhibited pyknotic nuclei, which is characteristic of apoptosis. In conclusion, PPP enhanced sorafenib-mediated suppression of proliferation and motility in HCC cells. Therefore, the combination of PPP and sorafenib may exert antitumor and antiangiogenic effects.

## Introduction

Vascular endothelial growth factor (VEGF) promotes angiogenesis in hepatocellular carcinoma (HCC). VEGF is upregulated in HCC as compared with surrounding non-HCC tissues (1); this upregulation has been correlated with advanced stage and poor outcome in HCC (2). Sorafenib, a multikinase inhibitor administered orally to HCC patients, targets the VEGF receptor, platelet-derived growth factor receptor and c-kit (3). Sorafenib treatment has been demonstrated to significantly prolong the survival times of HCC patients: 10.7 months as compared with 7.9 months in a placebo group (4).

However, one limitation of sorafenib treatment is the resistance of HCC to the reagent. Phosphatidyl-inositol (PI) 3 kinase and mitogen-activated protein (MAP) kinase are predominant downstream signaling pathways of VEGF that regulate cell proliferation (5). Although sorafenib inhibits the MAP kinase signaling pathway (6), the PI3 kinase signaling pathway is not affected, thereby resulting in HCC resistance (7). Another limitation of sorafenib is toxicity, which negatively affects the patient’s quality of life. For example, a high rate of dermatological adverse effects has been reported (4,8). However, administering a combination of sorafenib and other molecular targeting agents is expected to improve the efficacy and relieve particular adverse effects of the drug. For example, liver-specific microRNA-122 sensitizes tumors to the antitumor effects of sorafenib (9). However, a major limitation of microRNA is that the effects depend on transfection efficiency; untransfected cells are not affected. Therefore, small molecule inhibitors are desirable as these inhibitors affect the majority of cells.

Insulin-like growth factor (IGF)-1 is a hormone that is expressed abundantly in the fetus and exerts an important role in fetal growth and development. Inhibiting the IGF-1 signaling pathway in cancer therapy may have no adverse effects, since IGF-1 concentrations are reduced following birth (10). Picropodophyllin (PPP) is a specific inhibitor of the IGF-1 receptor (IGF-1R), which is involved in tumor cell growth (11,12). PPP has been shown to successfully suppress the proliferation of HCC and hepatoblastoma cells (13,14).

Therefore, in the present study, the proliferation and motility of HCC cells that had been treated with a combination of PPP and sorafenib were analyzed. Normal human umbilical vein endothelial cells (HUVECs) were also used to assess angiogenesis following drug treatment.

## Materials and methods

### Cell culture

HLF and PLC/PRF/5 HCC lines were purchased from the RIKEN cell bank (RIKEN Life Science Center, Tsukuba, Japan) and cultured in Dulbecco’s modified Eagle’s medium (DMEM; Sigma-Aldrich, St. Louis, MO, USA) supplemented with 10% fetal bovine serum (Life Technologies, Grand Island, NY, USA). HUVECs (Lonza, Basel, Switzerland) were cultured in EGM™-2 BulletKit™ (Lonza) following the manufacturer’s instructions. The cultured cells were incubated in 5% carbon dioxide at 37°C in a humidified chamber. Hematoxylin and eosin (H&E) staining was performed on cells grown in four-well chambers (Becton Dickinson, Franklin Lakes, NJ, USA) after 48 h of incubation.

### Cell proliferation assay

The HUVEC cells were trypsinized, harvested and plated onto 96-well flat-bottom plates (Asahi Techno Glass, Funabashi, Japan) at a density of 1,000 cells per well. Following 24 h of culture, sorafenib (JS Research Chemicals Trading e.Kfm, Wedel, Germany) or PPP (Wako Pure Chemical Industries, Ltd., Osaka, Japan) was added to the medium. After 72 h of incubation, a 3-(4,5-dimethylthiazol-2-yl)-5-(3-carboxymethoxyphenyl)-2-(4-sulfophenyl)-2H-tetrazolium inner salt (MTS) assay was performed following the manufacturer’s instructions (Promega Corporation, Madison, WI, USA). The MTS was bio-reduced by the cells into a colored formazan product, the absorbance of which was analyzed at a wavelength of 490 nm using an iMark microplate reader (Bio-Rad, Hercules, CA, USA).

### Scratch assay

The HUVEC cells were injured using a sterile 200-μm pipette tip at 24 h after plating into four-well chambers; the cells were then stained with H&E after 48 h (15). The distance between the scratched line and the growing edge of the cells was measured at five points.

### Statistical analysis

One-way analysis of variance was utilized for statistical analysis using JMP10.0.2 (SAS Institute, Cary, NC, USA). P<0.05 was considered to indicate a statistically significant difference.

## Results

### MTS assay

**Figure 1:**
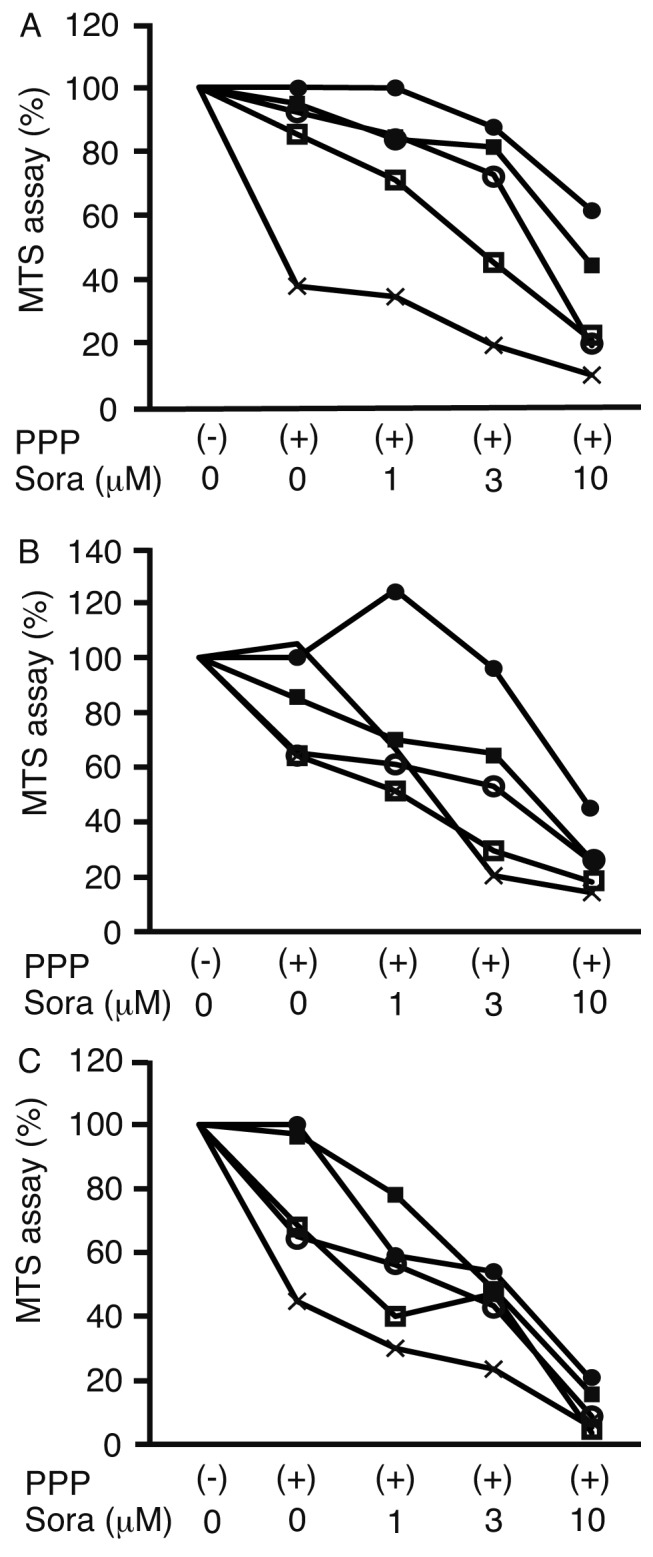
Cell proliferation assay. A 3-(4,5-dimethylthiazol-2-yl)-5-(3-carboxymethoxyphenyl)-2-(4-sulfophenyl)-2H-tetrazolium inner salt assay was performed following the addition of picropodophyllin (PPP) and/or sorafenib (Sora) to (A) HLF, (B) PLC/PRL/F and (C) normal human umbilical vein endothelial cells, and the cell proliferation rate is presented as the percentage of the untreated cell proliferation rate. ●, 0 μM PPP; ■, 0.02 μM PPP; ○, 0.06 μM PPP; □, 0.2 μM PPP; ×, 0.6 μM PPP; (−), without PPP; (+), with PPP, n=3.

The synergistic suppression of cell proliferation by PPP and sorafenib was analyzed using an MTS assay. The proliferation of HLF cells following treatment with 10 μM sorafenib and 0, 0.02, 0.06, 0.2 and 0.6 μM PPP was suppressed to 61.4±1.0, 44.3±14.0, 19.2±12.8, 20.9±6.3 and 10.3±6.7%, respectively, of the proliferation observed in the untreated control cells (Fig. 1A). Similarly, the proliferation of PLC/PRF/5 cells was suppressed to 44.2±0.1, 26.3±6.9, 25.5±8.3, 18.1±6.2 and 14.2±8.6% of control cell proliferation following treatment with 10 μM sorafenib and 0, 0.02, 0.06, 0.2 and 0.6 μM PPP, respectively (Fig. 1B). The proliferation of HUVECs was suppressed to 19.8±0.1, 15.6±2.9, 8.7±5.7, 3.5±1.8 and 5.4±3.8% control cell proliferation using the same respective treatments (Fig. 1C). Therefore, PPP inhibited cell proliferation in a dose-dependent manner.

Tables I and II show the raw data obtained from the MTS assays in HLF and PLC/RRF/5 cells, respectively. The combination of sorafenib and PPP suppressed cell proliferation more efficiently than 10 μM sorafenib alone. The initial aim was to reduce the concentration of sorafenib and, therefore, the effects of 3 μM sorafenib were analyzed. The combination of 3 μM sorafenib and 0.2 or 0.6 μM PPP suppressed the proliferation of HLF and PLC/PRF/5 cells more effectively than 10 μM sorafenib alone. Therefore, the combination of 3 μM sorafenib and 0.2 μM PPP was used for subsequent experiments.

### H&E staining

**Figure 2:**
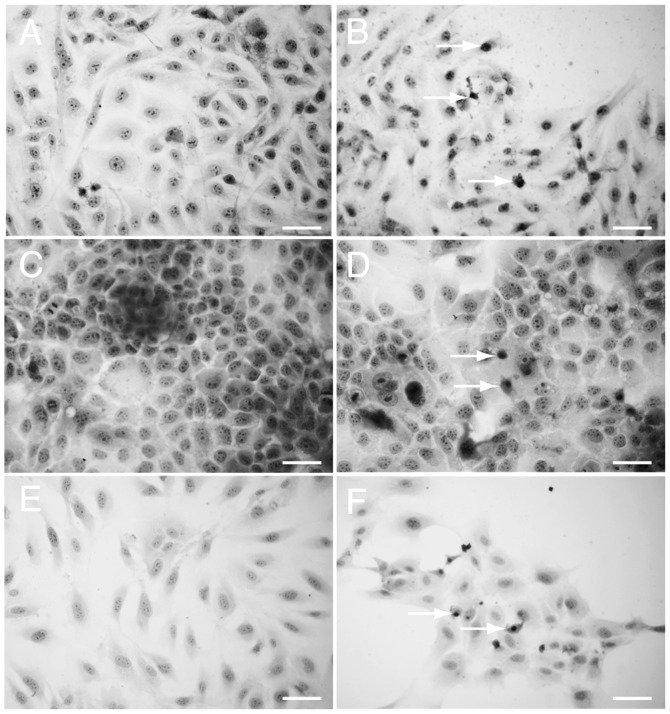
Hematoxylin and eosin staining. (A and B) HLF, (C and D) PLC/PRF/5 and (E and F) normal human umbilical vein endothelial cells were cultured on chamber slides. The cells were stained using hematoxylin and eosin following two days incubation with (B, D and F) or without (A, C and E) the addition of picropodophyllin (0.2 μM) and sorafenib (3 μM). Arrow, apoptotic cells with pyknotic nuclei; original magnification, ×400; scale bar, 50 μm.

HLF (Fig. 2A and B), PLC/PRF/5 (Fig. 2C and D) and HUVEC (Fig. 2E and F) cells were stained with H&E to assess the morphological changes following drug treatment. The cells that had been treated with 3 μM sorafenib and 0.2 μM PPP exhibited pyknotic nuclei, which is a characteristic of apoptotic cells (Fig. 2B, D and F). Pyknotic nuclei were not observed in the untreated cells (Fig. 2A, C and E).

### Scratch assay

**Figure 3:**
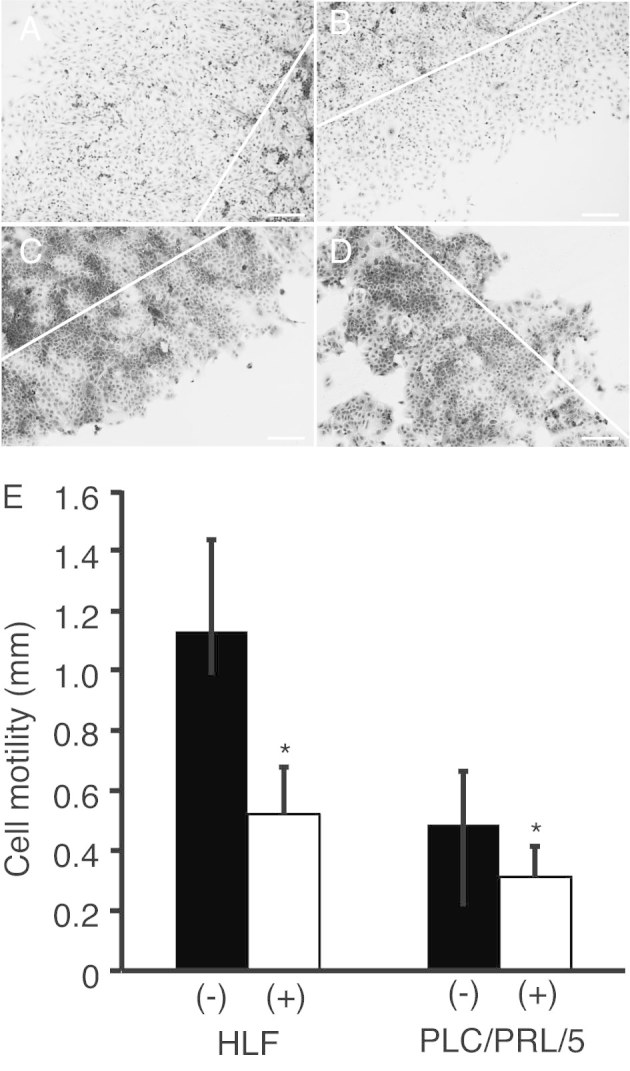
Scratch assay. (A and B) HLF or (C and D) PLC/PRL/5 cells were cultured on chamber slides. The cells were scratched using a 200-μl pipette tip (solid line), and treated with (B and D) or without (A and C) picropodophyllin (0.2 μM) and sorafenib (3 μM). (E) The distance between the scratched line and the growing edge of the cells was measured. Original magnification, ×100; scale bar, 200 μm; error bars indicate standard deviations. (−), cells cultured without picropodophyllin or sorafenib; (+), cells cultured with picropodophyllin and sorafenib. ^*^P<0.05, average distance of cells cultured with picropodophyllin and sorafenib compared with cells without picropodophyllin or sorafenib (n=3).

The motility of HLF and PLC/PRF/5 cells was analyzed using a scratch assay (Fig. 3A–D). The distance between the scratched line and the growing edge of the cells was measured for cells with (Fig. 3B and D) or without (Fig. 3A and C) treatment with 3 μM sorafenib and 0.2 μM PPP. Cell motility was significantly suppressed by the treatment, as compared with that of the control (P<0.05; Fig. 3E).

## Discussion

In the present study, PPP enhanced sorafenib-induced suppression of proliferation and motility in HCC cells. NVP-AEW541, another IGF-1R inhibitor, and sorafenib were previously shown to suppress cell proliferation and induce apoptosis synergistically (16). These data suggest that IGF-1R inhibitors and sorafenib suppress cell proliferation synergistically. Sorafenib upregulates IGF-1R and increases Akt (Ser473) phosphorylation (17,18). This suggests that sorafenib may activate signaling pathways downstream of IGF-1R; thus, treating HCC cells with IGF-1R inhibitors and sorafenib is feasible.

Cell motility may be used to indicate invasion and metastasis (19). The invasiveness of HCC cells has previously been revealed to be suppressed by PPP and sorafenib individually (14,20). However, to the best of our knowledge, the effects of the two drugs in combination have not been examined. In the present study, the effect of a combination of PPP and sorafenib on cell motility was assessed. The data clearly indicate that PPP and sorafenib suppressed the motility of HCC cells synergistically. This suggests that the combination of these two drugs may inhibit HCC metastasis to distant organs.

Suppressing angiogenesis is a predominant mechanism of the antitumor effects of sorafenib (21). Similarly, inhibiting IGF-1R was shown to suppress the proliferation of HUVECs and induce apoptosis (22). The present study clearly demonstrated that the combination of sorafenib and PPP markedly suppressed proliferation and induced apoptosis in HUVECs. This suggests that the combination of PPP and sorafenib may more effectively suppress angiogenesis.

The combination of 1 μM NVP-AEW541 and 10 μM sorafenib has been previously demonstrated to suppress cell proliferation more effectively than 10 μM sorafenib alone (16). However, the combination of NVP-AEW541 and concentrations of sorafenib lower than10 μM has not been investigated. In the present study, the combination of 0.2 μM PPP and 3 μM sorafenib decreased cell proliferation more efficiently than 10 μM sorafenib alone. This suggests that using co-treatment with an IGF-1R inhibitor may allow the effective dose of sorafenib to be reduced, which may lower the risk of adverse effects. Nevertheless, the combination of PPP and sorafenib may cause different adverse events. Thus, future studies that analyze, through western blotting, the signaling pathways that are altered by co-treatment are required.

In conclusion, in the present study, PPP enhanced sorafenib-induced suppression of proliferation and motility in HCC cells. Therefore, the combination of PPP and sorafenib may exert antitumor and antiangiogenic effects.

## Figures and Tables

**Table I:** Cell proliferation assay in HLF cells.

	Sorafenib (μM)			
	
	0	1	3	10
PPP (μM)				
0.00	100	99.8±7.8	87.5±5.6	61.6±1.0
0.02	94.9±5.2	83.7±12.4	81.3±9.5	44.3±14.0^a^
0.06	92.2±7.9	85.4±7.2	72.7±10.3	19.2±7.8^a^
0.20	85.3±12.4	71.3±15.3	45.2±6.2^a^	20.9±6.3^a^
0.60	37.8±2.9^a^	34.5±8.1^a^	19.3±8.7^a^	10.0±6.7^a^

Data are presented as the mean ± standard deviation cell proliferation as a percentage of the untreated control cell proliferation.

a Variables lower than those treated with sorafenib at 10 μM alone, n=3.

PPP, picropodophyllin.

**Table II:** Cell proliferation assay in PLC/PRF/5 cells.

	Sorafenib (μM)			
	
	0	1	3	10
PPP (μM)				
0.00	100	124.6±30.1	95.8±10.7	44.2±0.1
0.02	85.2±9.5	69.7±9.8	64.6±13.2	26.3±6.9^a^
0.06	65.2±6.9	61.0±13.2	53.4±12.5	25.5±8.3^a^
0.20	105.4±7.2	66.7±11.4	20.4±9.7^a^	14.2±8.6^a^
0.60	64.2±8.1	51.3±4.1	29.5±6.9^a^	18.1±6.2^a^

Data are presented as the mean ± standard deviation cell proliferation as a percentage of the untreated control cell proliferation.

a Variables lower than those with sorafenib at 10 μM alone, n=3.

PPP, picropodophyllin.
